# Supplementary material for: Loss of CFHR5 function reduces the risk for age-related macular degeneration
Source: Nat Commun. 2025 Jul 1;16:5766. doi: 10.1038/s41467-025-61193-3 (PMC12217273; doi:10.1038/s41467-025-61193-3)

SUPPLEMENTARY INFORMATION

to:

Loss of *CFHR5* function reduces the risk for age-related macular degeneration

Mary Pat Reeve[1,2,3,*], Stephanie Loomis[4,*], Eija Nissilä[5], Tom Soare[6], Tobias Rausch[7], Zhili Zheng[2,3], Pietro Della Briotta Parolo[2,3], Daniel Ben-Isvy[3,8,9], Elias Aho[5], Emilia Cesetti[5,10], Yoko Okunuki[4], Helen McLaughlin[4], Johanna Mäkelä[11], FinnGen, Mitja Kurki[1,2,3], Michael E. Talkowski[3,8], Jan O. Korbel[7], Kip Connor[4], Seppo Meri[5], Mark J. Daly[1,2,3,$,#], Heiko Runz[1,4,6, 7,$,#]

1. Institute for Molecular Medicine Finland (FIMM), University of Helsinki, Helsinki, Finland
2. Analytic and Translational Genetics Unit, Department of Medicine, Massachusetts General Hospital, Boston, MA, USA
3. Program in Medical and Population Genetics, Broad Institute of Harvard and MIT, Cambridge, MA, USA
4. Research and Development, Biogen Inc., Cambridge, MA, USA
5. Department of Bacteriology and Immunology, Translational Immunology Research Program, University of Helsinki, Helsinki, Finland
6. insitro Inc., South San Francisco, CA, USA
7. European Molecular Biological Laboratories (EMBL), Heidelberg, Germany
8. Center for Genomic Medicine, Massachusetts General Hospital, Boston, MA, USA
9. Division of Medical Sciences, Harvard Medical School, Boston, MA, USA
10. Department of Biomedical Sciences, Humanitas University, Milan, Italy
11. Finnish Biobank Cooperative (FinBB), Turku, Finland

* joint contribution
[$] joint supervision

[#] communication: heiko.runz@gmail.com or mjdaly@broadinstitute.org

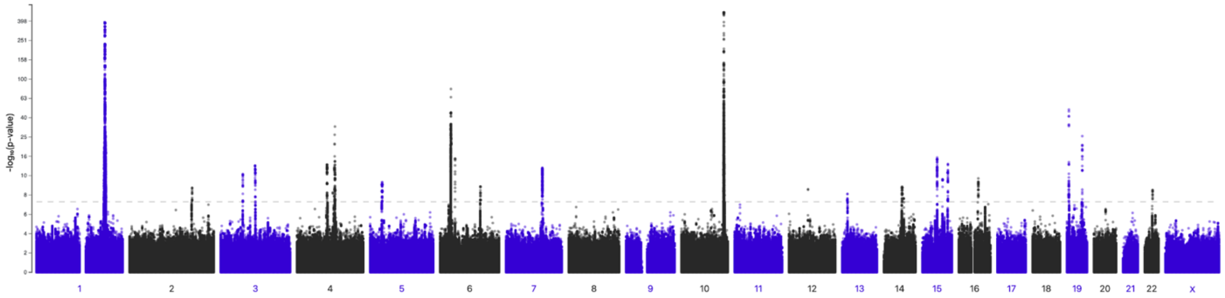

Supplementary Figure 1. Manhattan plot of genome-wide association analysis for age-related macular degeneration. GWAS summary statistics are derived from 12,495 cases and 461,686 controls in FinnGen (DF12; see also https://r12.finngen.fi/pheno/H7_AMD). AMD cases are defined has having at least one entry with the diagnostic code H7_AMD, reflecting both wet and dry AMD. (x-axis), genomic position of genetic variant; (y-axis), -log10(P-value) of tested genetic variant. Dashed horizontal line indicates genome-wide significance threshold ($P<5x10^{-8}$).

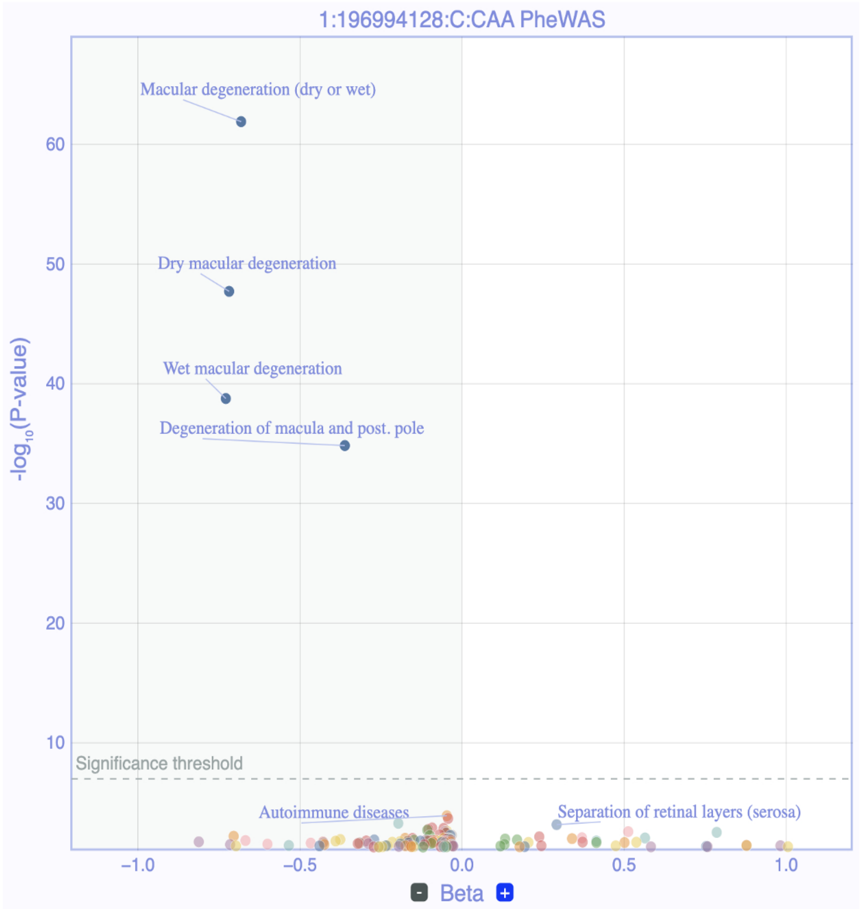

Supplementary Figure 2. Phenome-scan of $\mathit{CFHR5}_{fs}$ frameshift variant. LAVAA plot for the Finnish-enriched *CFHR5* frameshift variant p.Glu163insAA (chr1:196994128:C:CAA, rs565457964) in FinnGen (DF12) against 2,408 FinnGen phenotypes (see also https://r12.finngen.fi/variant/1:196994128-C-CAA). Each dot represents association signals for one respective phenotype. Phenotypes with associations meeting genome-wide significance and of putative relevance for drug repositioning and safety are named. (x-axis), beta of association signal; (y-axis), -log10(P-value) of tested genetic variant. Dashed horizontal line reflects genome-wide significance threshold ($P<5x10^{-8}$).

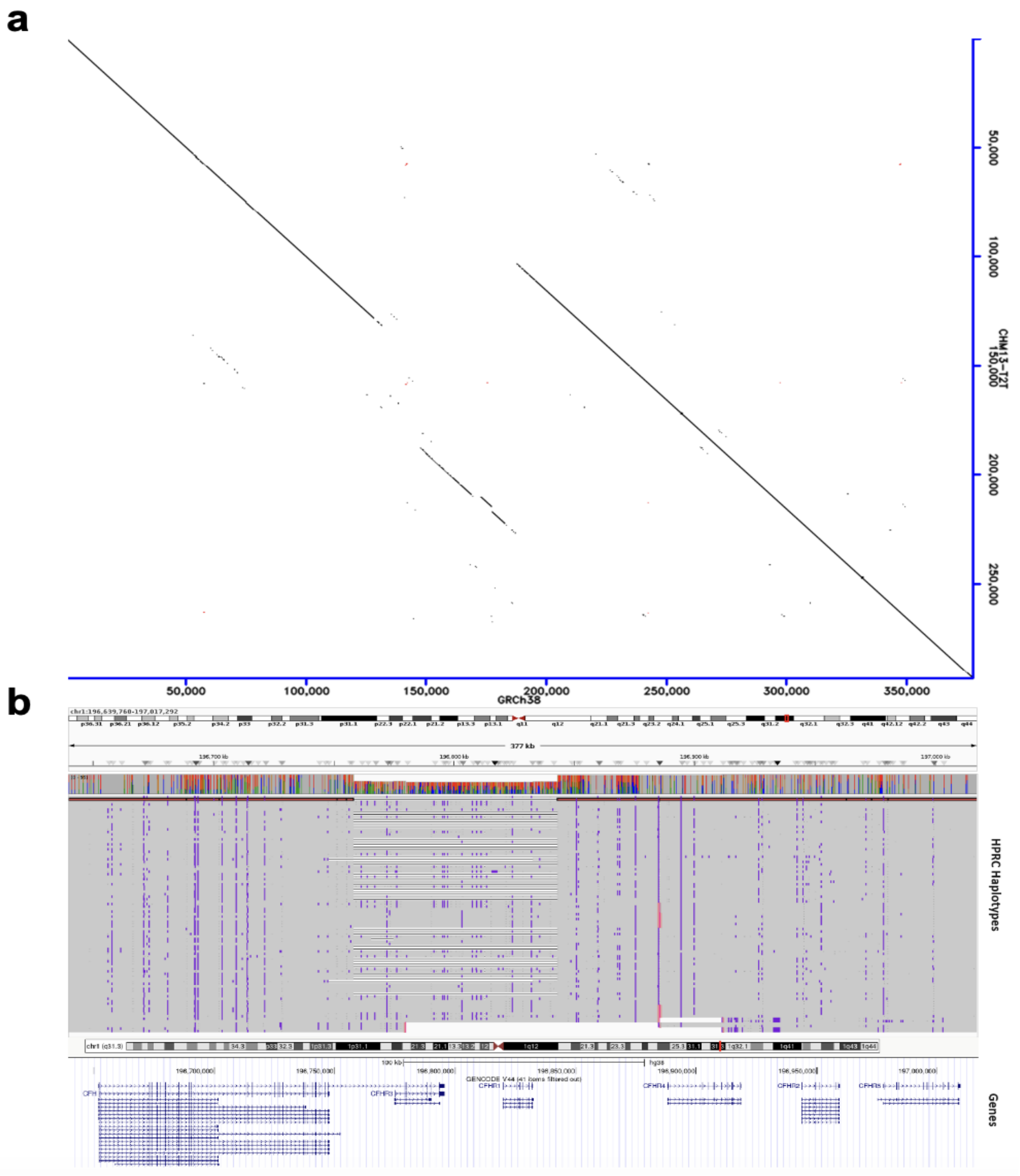

Supplementary Figure 3. Haplotype structure of the *CFH* region as mapped through phased long-read assemblies. (a) Dot plot of the chromosomal region chr1:196,639,760-197,017,292 in GRCh38 assembly (x-axis) compared to the corresponding region chr1:195,986,103-196,279,118 in CHM13-T2T assembly (y-axis). The center of the region reveals a previously described prevalent deletion of ~91kb (HGSV_15069) present in 27.2% of sequenced individuals. **(b)** IGV view of the same region with all Human Pangenome Reference Consortium (HPRC) haplotypes (48 samples) aligned to GRCh38. The CHM13-T2T haplotype is included in this view and highlighted in red. Annotation of protein-coding gene regions reveals that the deletion encompasses *CFHR3* and *CFHR1* genes.

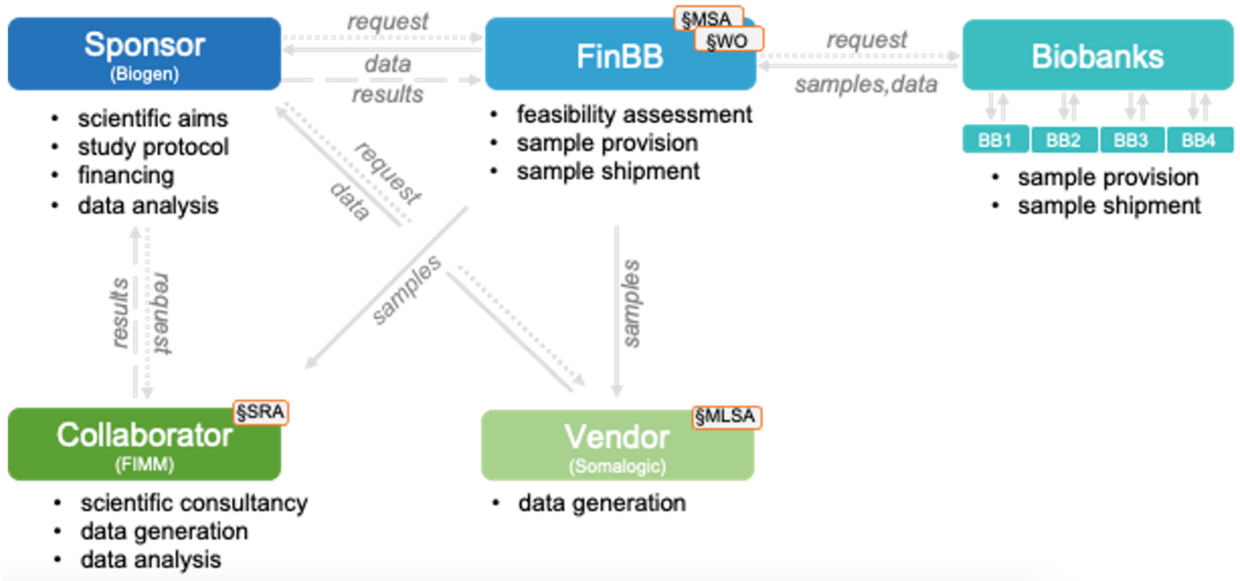

Supplementary Figure 4. Workflow for FinnGen sample recall study from Finnish biobanks. Scientific project definition and drafting of a concrete study protocol were facilitated by the Finnish Biobank Cooperative (FinBB) who conducted feasibility assessments in four selected Finnish biobanks. Available samples from cases and controls were matched based on electronic health data according to the sponsor's in- and exclusion criteria and shipped to a vendor (Somalogic) and academic collaborators at the Finnish Institute for Molecular Medicine (FIMM) for data generation. Data was jointly analyzed by the study's sponsor and academic collaborators. Study realization required independent contracting with FinBB (MSA, master services agreement; WO, work order), the vendor (MLSA, master license and services agreement) and the academic partner (SRA, sponsored research agreement).

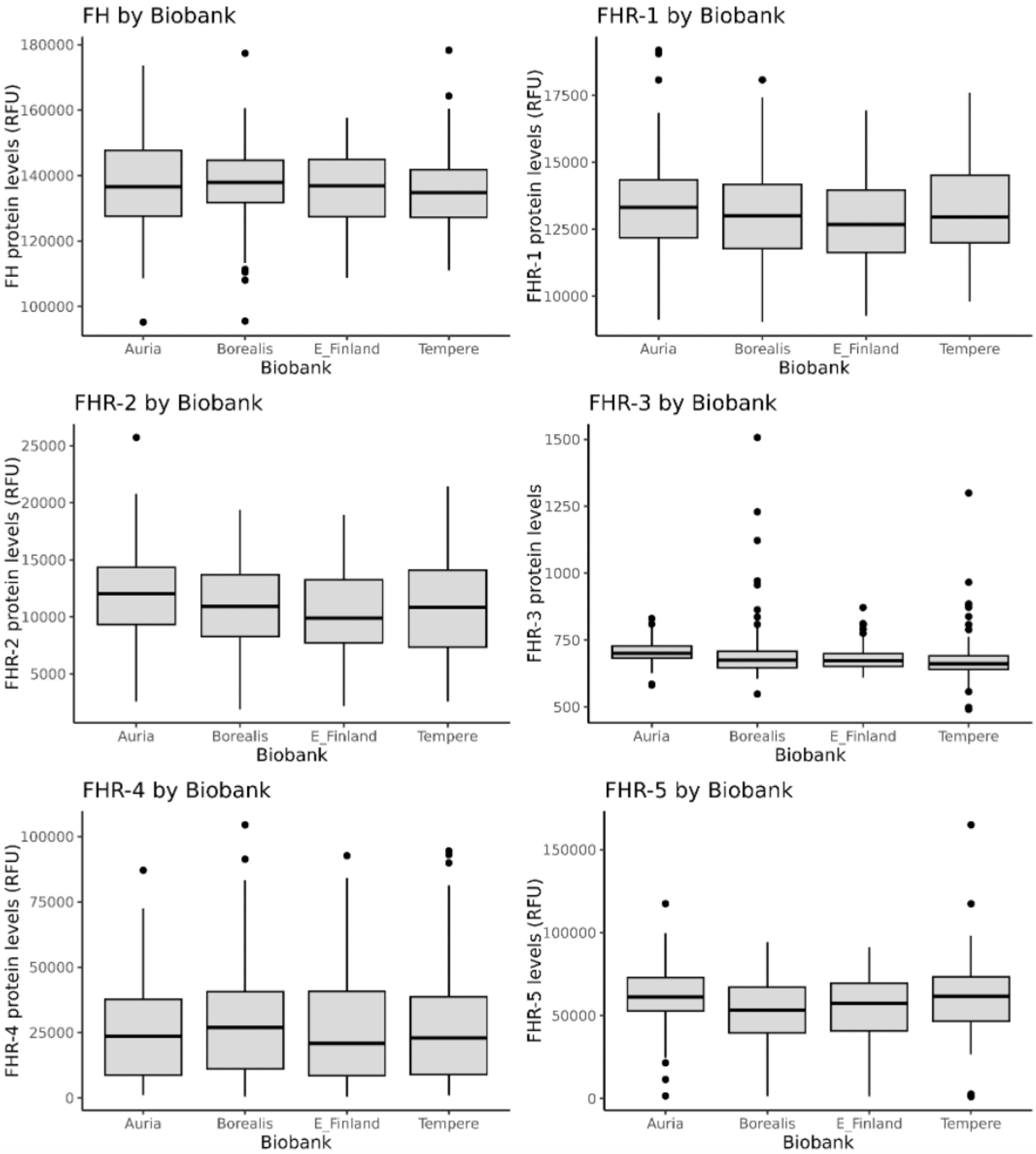

Supplementary Figure 5. Serum levels of FH and FHR1-5 proteins by biobank. Relative protein of Complement factor H (FH) and Complement factor H related factors FHR1-5 as measured by representative somamers on the SomaScan platform from serum samples of 399 FinnGen participants recalled from four Finnish biobanks. (x-axis) reflects participating biobank (see Supplementary Data 11 and 12). Univariate linear regression analyses were performed using R. In the box plots, the center line represents the median, the box limits represent the interquartile range (IQR) and the whiskers indicate the minimum and maximum values. E_Finland, Biobank of Eastern Finland. RFU, relative fluorescence units.

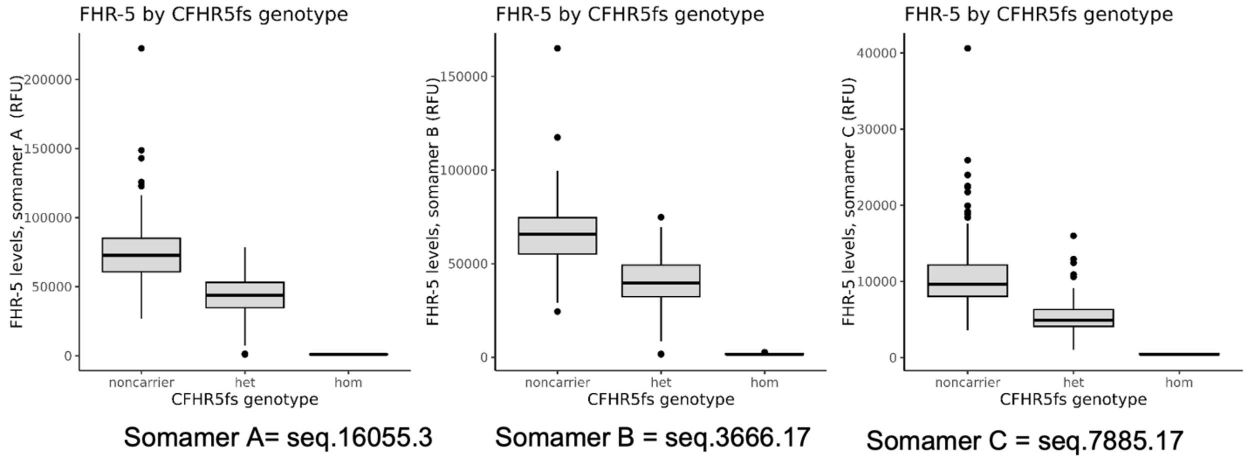

Supplementary Figure 6. FHR-5 levels by *CFHR5*$_{fs}$ status for three different FHR-5 somamers. Relative protein levels of Complement factor H related factor 5 (FHR5) as measured by three different somamers (A-C) on the SomaScan platform from serum samples of 399 FinnGen participants recalled from four Finnish biobanks. (x-axis) reflects *CFHR5*$_{fs}$ carrier status (see Supplementary Data 11, 13 and 14). Univariate linear regression analyses were performed using R. In the box plots, the center line represents the median, the box limits represent the interquartile range (IQR) and the whiskers indicate the minimum and maximum values. het, heterozygotes; hom, homozygotes. RFU, relative fluorescence units.

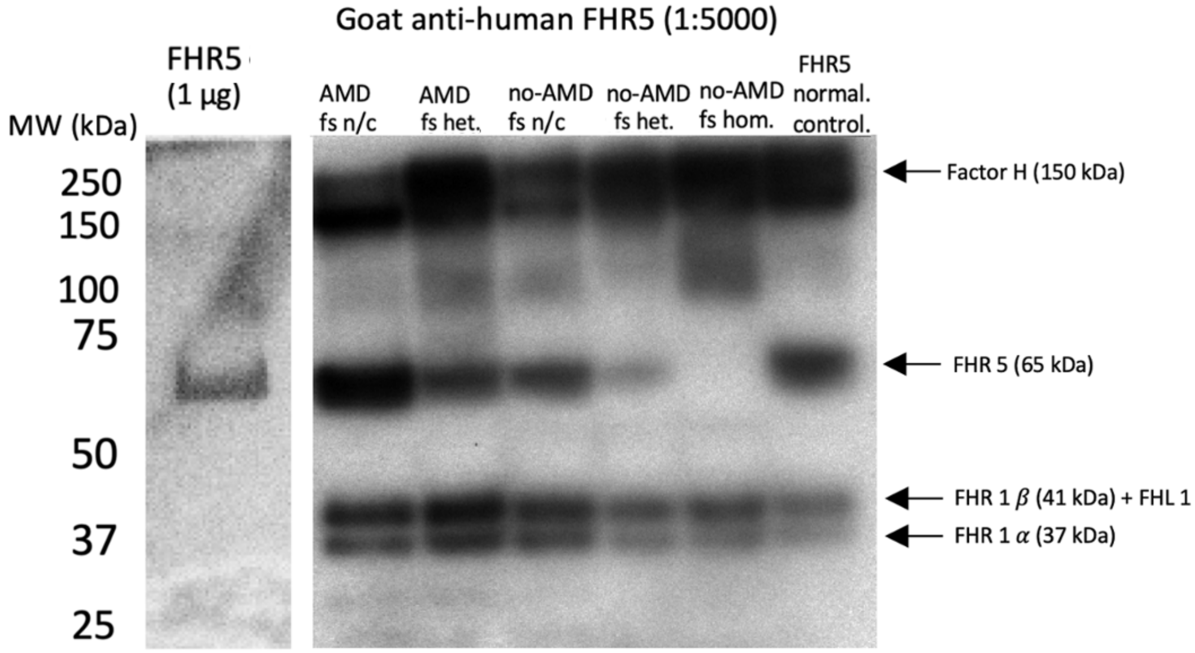

Supplementary Figure 7. FHR-5 levels by *CFHR5*$_{fs}$ status as detected by Western Blot. Left, purified FHR-5 protein (1µg/lane). Right, serum samples from recall-study participants with indicated genotypes with and without (no-AMD) a registry-based diagnosis of AMD. n/c, non-carrier; fs het., heterozygous for *CFHR5*$_{fs}$; fs hom., homozygous for *CFHR5*$_{fs}$. A control from an independent healthy volunteer is on the right-most lane. Note that due to high structural similarities, the polyclonal antibody against FHR-5 also reacts with Factor H and some other closely related proteins.

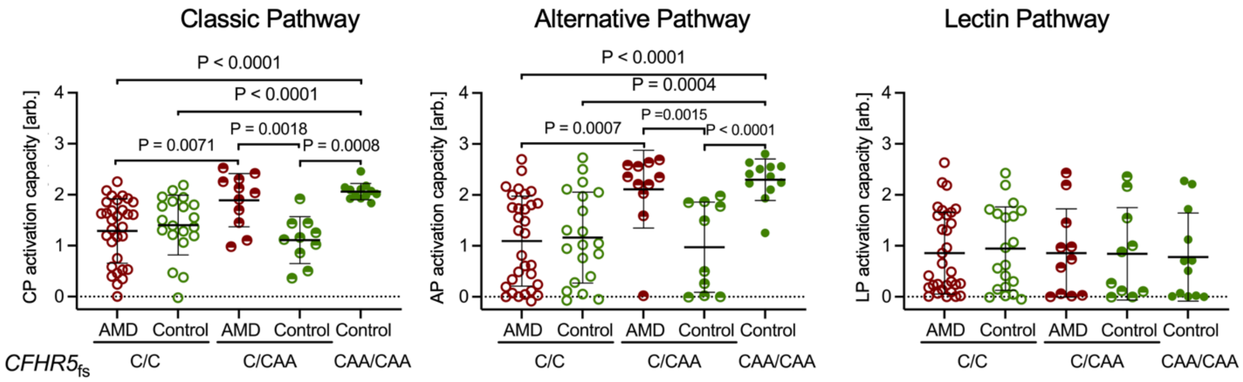

Supplementary Figure 8. Complement activity in *CFHR5*$_{fs}$ carriers relative to AMD status. Functional analysis of CP, AP, and LP activation capacity in serum samples from 84 recall study participants grouped into individuals with (n=40; red) and without (n=44; green) a registry-based diagnosis of AMD (see Supplementary Data 18). (x-axis) reflects carrier status for *CFHR5* frameshift variant p.Glu163insAA. C/C, non-carriers; C/CAA, heterozygotes; CAA/CAA, homozygotes. Statistical analyses were performed with the Mann-Whitney U-test. The Kruskal-Wallis test was applied for comparisons across all three groups. Each dot represents one individual. For each group the center line represents the median, the whiskers the standard deviation. AU, arbitrary units.

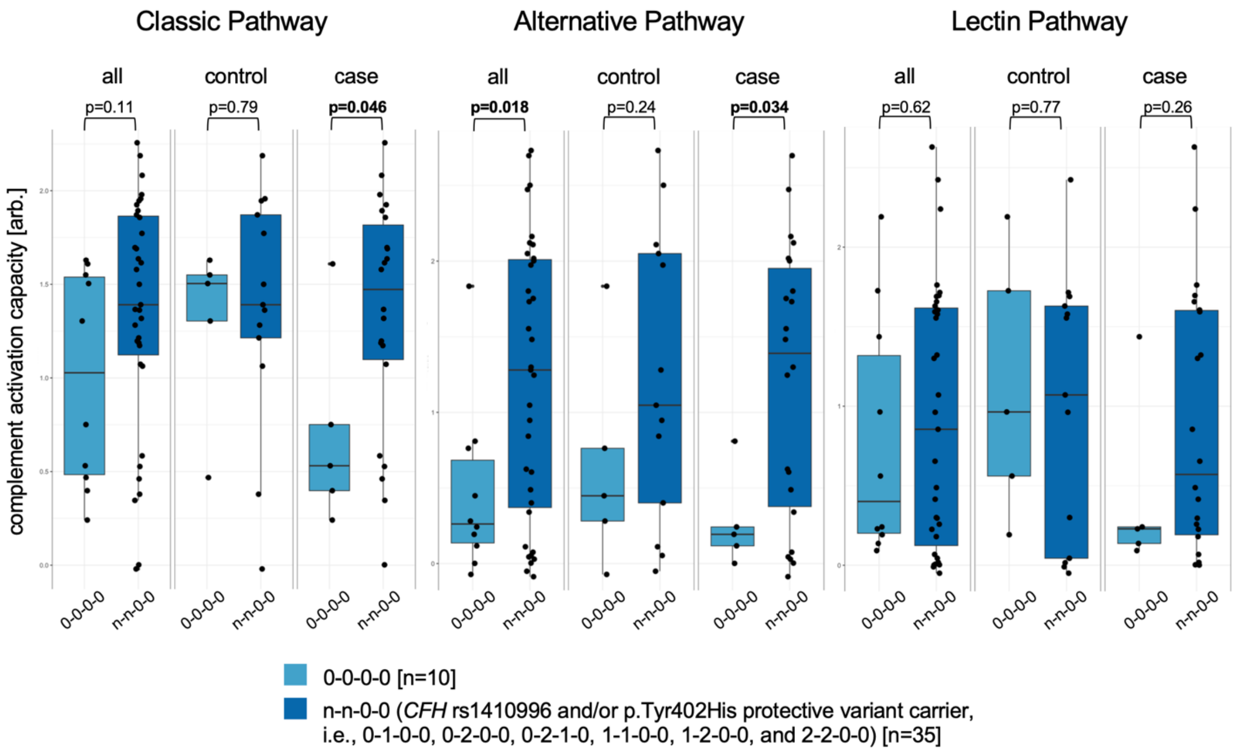

Supplementary Figure 9. Complement activation capacity in *CFH* protective variant carriers relative to non-carriers. Functional analysis of CP, AP and LP activation capacity in serum samples from 45 recall study participants grouped into carriers (n=35, dark blue) or non-carriers (n=10; 0-0-0-0, light blue) of AMD protective *CFH* alleles at rs1410996 and p.Tyr402His (n-n-0-0) (see Supplementary Data 18). Recall study participants carrying either the *CFHR5* frameshift variant p.Glu163insAA of the *CFHR5* p.Gly278Ser were excluded from this analysis. Statistical analyses were conducted with a 2-sided, 2-tailed Student’s T-test. Each dot represents one individual. For each group the center line represents the median, the whiskers the standard deviation. arb., arbitrary units.

Appendix: Original Western Blots to Supplementary Figure 7

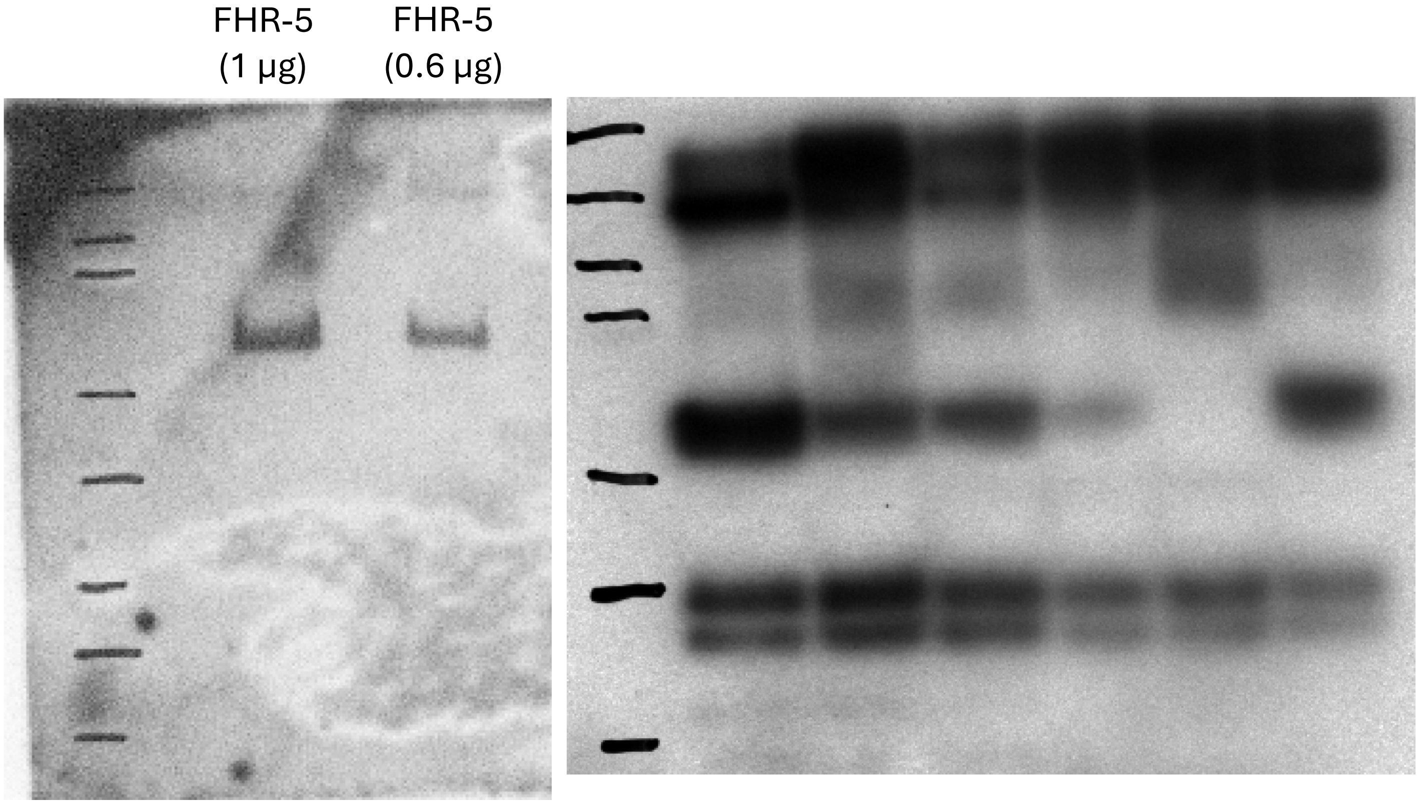

Supplement: Supplementary file 1 — Supplementary Information [file 41467_2025_61193_MOESM1_ESM.pdf]
