# Supplementary material for: Loss of CFHR5 function reduces the risk for age-related macular degeneration
Source: Nat Commun. 2025 Jul 1;16:5766. doi: 10.1038/s41467-025-61193-3 (PMC12217273; doi:10.1038/s41467-025-61193-3)
Supplement: Supplementary file 3 — Reporting Summary [file 41467_2025_61193_MOESM3_ESM.pdf]

Corresponding author(s): Heiko Runz, Mark Daly

Last updated by author(s): May 5, 2025

Reporting Summary

Nature Portfolio wishes to improve the reproducibility of the work that we publish. This form provides structure for consistency and transparency in reporting. For further information on Nature Portfolio policies, see our Editorial Policies and the Editorial Policy Checklist.

Please do not complete any field with "not applicable" or n/a. Refer to the help text for what text to use if an item is not relevant to your study.
For final submission: please carefully check your responses for accuracy; you will not be able to make changes later.

Statistics

For all statistical analyses, confirm that the following items are present in the figure legend, table legend, main text, or Methods section.

n/a	Confirmed	
☐	☒	The exact sample size (*n*) for each experimental group/condition, given as a discrete number and unit of measurement
☐	☒	A statement on whether measurements were taken from distinct samples or whether the same sample was measured repeatedly
☐	☒	The statistical test(s) used AND whether they are one- or two-sided<br>*Only common tests should be described solely by name; describe more complex techniques in the Methods section.*
☐	☒	A description of all covariates tested
☐	☒	A description of any assumptions or corrections, such as tests of normality and adjustment for multiple comparisons
☐	☒	A full description of the statistical parameters including central tendency (e.g. means) or other basic estimates (e.g. regression coefficient) AND variation (e.g. standard deviation) or associated estimates of uncertainty (e.g. confidence intervals)
☐	☒	For null hypothesis testing, the test statistic (e.g. *F*, *t*, *r*) with confidence intervals, effect sizes, degrees of freedom and *P* value noted<br>*Give P values as exact values whenever suitable.*
☒	☐	For Bayesian analysis, information on the choice of priors and Markov chain Monte Carlo settings
☒	☐	For hierarchical and complex designs, identification of the appropriate level for tests and full reporting of outcomes
☒	☐	Estimates of effect sizes (e.g. Cohen's *d*, Pearson's *r*), indicating how they were calculated

Our web collection on statistics for biologists contains articles on many of the points above.

Software and code

Policy information about availability of computer code

Data collection

FG data analysis pipelines are freely available from https://github.com/FINNGEN/. The FinnGen Handbook, https://finngen.gitbook.io/documentation/, contains a detailed description of data production and analysis, including code used to run the analyses described in this manuscript.

Data analysis

For manuscripts utilizing custom algorithms or software that are central to the research but not yet described in published literature, software must be made available to editors and reviewers. We strongly encourage code deposition in a community repository (e.g. GitHub). See the Nature Portfolio guidelines for submitting code & software for further information.

Data

Policy information about availability of data

All manuscripts must include a data availability statement. This statement should provide the following information, where applicable:

- Accession codes, unique identifiers, or web links for publicly available datasets
- A description of any restrictions on data availability
- For clinical datasets or third party data, please ensure that the statement adheres to our policy

FG summary association results are available for download at https://www.finngen.fi/en/access_results and can be explored in a public results browser (https://r12.finngen.fi). All analyses in this manuscript which rely on variants that were directly interrogated through chip-based genotyping with the FG array or imputed rely on FG data freeze 12, which was publicly released in November 2024. Individual-level data from FG participants can be accessed by application to the Finnish Biobank Cooperative FinBB (https://finbb.fi/en/).

Research involving human participants, their data, or biological material

Policy information about studies with human participants or human data. See also policy information about sex, gender (identity/presentation), and sexual orientation and race, ethnicity and racism.

Reporting on sex and gender	Sex and gender are reported for study participants. There were no sex biases with regards to study inclusion or analysis.
Reporting on race, ethnicity, or other socially relevant groupings	Ethnicity is reported for all study participants. The majority of individuals analyses are of Finnish origin and participants of the FinnGen research project. Among this sample there were no biases with regards to ethnicity for study inclusion or analysis.
Population characteristics	see https://www.finngen.fi/en/node/2635 and https://risteys.finngen.fi
Recruitment	see https://www.finngen.fi/en
Ethics oversight	see https://www.finngen.fi/en and extensive FinnGen DF12 Ethics Statement provided in the manuscript

Note that full information on the approval of the study protocol must also be provided in the manuscript.

Field-specific reporting

Please select the one below that is the best fit for your research. If you are not sure, read the appropriate sections before making your selection.

[X] Life sciences [ ] Behavioural & social sciences [ ] Ecological, evolutionary & environmental sciences

For a reference copy of the document with all sections, see nature.com/documents/nr-reporting-summary-flat.pdf

Life sciences study design

All studies must disclose on these points even when the disclosure is negative.

Sample size	Overall FinnGen cohort used: n=500,348; among these FinnGen AMD recall study participants: n=400
Data exclusions	For details on FinnGen data and data in/exlusion creteria, see https://www.finngen.fi/en/node/2635
Replication	The majority of attempts at replicate FinnGen findings from this study in UK Biobank were successful. For potential reasons why few findings might not have replicated (for instance due to differences in target populations and study designs, see also https://www.ukbiobank.ac.uk/media/3sbeknnz/ukbiobank_protocol.pdf)
Randomization	AMD cases/controls were selected based on individual-level phenotype data in the 4 participating biobanks; for in/exclusion criteria see manuscript
Blinding	The investigators were blinded from which samples from a larger pool were selected for the recall study. For the complement assay, the experimentor was blinded from the genotype/haplotype status if tested samples.

Behavioural & social sciences study design

All studies must disclose on these points even when the disclosure is negative.

Study description	
Research sample	
Sampling strategy	
Data collection	
Timing	
Data exclusions	
Non-participation	
Randomization	

Ecological, evolutionary & environmental sciences study design

All studies must disclose on these points even when the disclosure is negative.

Study description

Research sample

Sampling strategy

Data collection

Timing and spatial scale

Data exclusions

Reproducibility

Randomization

Blinding

Did the study involve field work? ☐ Yes ☐ No

Field work, collection and transport

Field conditions

Location

Access & import/export

Disturbance

Reporting for specific materials, systems and methods

We require information from authors about some types of materials, experimental systems and methods used in many studies. Here, indicate whether each material, system or method listed is relevant to your study. If you are not sure if a list item applies to your research, read the appropriate section before selecting a response.

Materials & experimental systems

n/a	Involved in the study	
☐	X	Antibodies
X	☐	Eukaryotic cell lines
X	☐	Palaeontology and archaeology
X	☐	Animals and other organisms
X	☐	Clinical data
☐	X	Dual use research of concern
X	☐	Plants

Methods

n/a	Involved in the study	
X	☐	ChIP-seq
X	☐	Flow cytometry
☐	X	MRI-based neuroimaging

Antibodies

Antibodies used

Validation

Eukaryotic cell lines

Policy information about cell lines and Sex and Gender in Research

Cell line source(s)	
Authentication	
Mycoplasma contamination	
Commonly misidentified lines (See ICLAC register)	

Palaeontology and Archaeology

Specimen provenance	
Specimen deposition	
Dating methods	

☐ Tick this box to confirm that the raw and calibrated dates are available in the paper or in Supplementary Information.

Ethics oversight	

Note that full information on the approval of the study protocol must also be provided in the manuscript.

Animals and other research organisms

Policy information about studies involving animals; ARRIVE guidelines recommended for reporting animal research, and Sex and Gender in Research

Laboratory animals	
Wild animals	
Reporting on sex	
Field-collected samples	
Ethics oversight	

Note that full information on the approval of the study protocol must also be provided in the manuscript.

Clinical data

Policy information about clinical studies

All manuscripts should comply with the ICMJE guidelines for publication of clinical research and a completed CONSORT checklist must be included with all submissions.

Clinical trial registration	
Study protocol	
Data collection	
Outcomes	

Dual use research of concern

Policy information about dual use research of concern

Hazards

Could the accidental, deliberate or reckless misuse of agents or technologies generated in the work, or the application of information presented in the manuscript, pose a threat to:

No	Yes	
[x]	[ ]	Public health
[x]	[ ]	National security
[x]	[ ]	Crops and/or livestock
[x]	[ ]	Ecosystems
[x]	[ ]	Any other significant area

Experiments of concern

Does the work involve any of these experiments of concern:

No	Yes	
[x]	[ ]	Demonstrate how to render a vaccine ineffective
[x]	[ ]	Confer resistance to therapeutically useful antibiotics or antiviral agents
[x]	[ ]	Enhance the virulence of a pathogen or render a nonpathogen virulent
[x]	[ ]	Increase transmissibility of a pathogen
[x]	[ ]	Alter the host range of a pathogen
[x]	[ ]	Enable evasion of diagnostic/detection modalities
[x]	[ ]	Enable the weaponization of a biological agent or toxin
[x]	[ ]	Any other potentially harmful combination of experiments and agents

Plants

Seed stocks	
Novel plant genotypes	
Authentication	

ChIP-seq

Data deposition

- [ ] Confirm that both raw and final processed data have been deposited in a public database such as GEO.
- [ ] Confirm that you have deposited or provided access to graph files (e.g. BED files) for the called peaks.

Data access links<br>*May remain private before publication.*	
Files in database submission	
Genome browser session<br>(e.g. UCSC)	

Methodology

Replicates	
Sequencing depth	
Antibodies	
Peak calling parameters	
Data quality	

Software

Flow Cytometry

Plots

Confirm that:

☐ The axis labels state the marker and fluorochrome used (e.g. CD4-FITC).

☐ The axis scales are clearly visible. Include numbers along axes only for bottom left plot of group (a 'group' is an analysis of identical markers).

☐ All plots are contour plots with outliers or pseudocolor plots.

☐ A numerical value for number of cells or percentage (with statistics) is provided.

Methodology

Sample preparation

Instrument

Software

Cell population abundance

Gating strategy

☐ Tick this box to confirm that a figure exemplifying the gating strategy is provided in the Supplementary Information.

Magnetic resonance imaging

Experimental design

Design type: Publicly released Optical Coherence Tomography (OCT) scans from UK Biobank. Analyses with UK Biobank data were conducted under the UKB project number 51766. Image data qc and analysis is provided in detail in Methods.

Design specifications

Behavioral performance measures

Imaging type(s)

Field strength

Sequence & imaging parameters

Area of acquisition

Diffusion MRI ☐ Used ☐ Not used

Preprocessing

Preprocessing software

Normalization

Normalization template

Noise and artifact removal

Volume censoring

Statistical modeling & inference

Model type and settings

Effect(s) tested

Specify type of analysis: ☐ Whole brain ☐ ROI-based ☐ Both

Statistic type for inference

(See Eklund et al. 2016)

Correction

Models & analysis

n/a	Involved in the study
☐	☐ Functional and/or effective connectivity
☐	☐ Graph analysis
☐	☐ Multivariate modeling or predictive analysis

Functional and/or effective connectivity

Graph analysis

Multivariate modeling and predictive analysis

This checklist template is licensed under a Creative Commons Attribution 4.0 International License, which permits use, sharing, adaptation, distribution and reproduction in any medium or format, as long as you give appropriate credit to the original author(s) and the source, provide a link to the Creative Commons license, and indicate if changes were made. The images or other third party material in this article are included in the article's Creative Commons license, unless indicated otherwise in a credit line to the material. If material is not included in the article's Creative Commons license and your intended use is not permitted by statutory regulation or exceeds the permitted use, you will need to obtain permission directly from the copyright holder. To view a copy of this license, visit http://creativecommons.org/licenses/by/4.0/
